# Enhancing biomechanical machine learning with limited data: generating realistic synthetic posture data using generative artificial intelligence

**DOI:** 10.3389/fbioe.2024.1350135

**Published:** 2024-02-14

**Authors:** Carlo Dindorf, Jonas Dully, Jürgen Konradi, Claudia Wolf, Stephan Becker, Steven Simon, Janine Huthwelker, Frederike Werthmann, Johanna Kniepert, Philipp Drees, Ulrich Betz, Michael Fröhlich

**Affiliations:** ^1^ Department of Sports Science, University of Kaiserslautern-Landau, Kaiserslautern, Germany; ^2^ Institute of Physical Therapy, Prevention and Rehabilitation, University Medical Centre, Johannes Gutenberg University Mainz, Mainz, Germany; ^3^ Department of Orthopedics and Trauma Surgery, University Medical Centre, Johannes Gutenberg University Mainz, Mainz, Germany

**Keywords:** machine learning, deep learning, spine, variational autoencoder, data augmentation, statistical parametric mapping

## Abstract

**Objective:** Biomechanical Machine Learning (ML) models, particularly deep-learning models, demonstrate the best performance when trained using extensive datasets. However, biomechanical data are frequently limited due to diverse challenges. Effective methods for augmenting data in developing ML models, specifically in the human posture domain, are scarce. Therefore, this study explored the feasibility of leveraging generative artificial intelligence (AI) to produce realistic synthetic posture data by utilizing three-dimensional posture data.

**Methods:** Data were collected from 338 subjects through surface topography. A Variational Autoencoder (VAE) architecture was employed to generate and evaluate synthetic posture data, examining its distinguishability from real data by domain experts, ML classifiers, and Statistical Parametric Mapping (SPM). The benefits of incorporating augmented posture data into the learning process were exemplified by a deep autoencoder (AE) for automated feature representation.

**Results:** Our findings highlight the challenge of differentiating synthetic data from real data for both experts and ML classifiers, underscoring the quality of synthetic data. This observation was also confirmed by SPM. By integrating synthetic data into AE training, the reconstruction error can be reduced compared to using only real data samples. Moreover, this study demonstrates the potential for reduced latent dimensions, while maintaining a reconstruction accuracy comparable to AEs trained exclusively on real data samples.

**Conclusion:** This study emphasizes the prospects of harnessing generative AI to enhance ML tasks in the biomechanics domain.

## 1 Introduction

Biomechanics, the study of human movement and its mechanical principles, holds great promise for advancing our understanding of human locomotion, aiding clinical diagnoses, and enhancing athletic performance (Barnes and Kilding, 2015; Ferreira et al., 2016; Ceyssens et al., 2019; Valamatos et al., 2022). In biomechanical data analysis, Artificial Intelligence (AI) and Machine Learning (ML) methods have gained traction (Halilaj et al., 2018; Phinyomark et al., 2018; Dindorf et al., 2022a), yielding promising results, such as in studies involving post-stroke patients (Lau et al., 2009) or Parkinson’s disease (Wahid et al., 2015). These approaches excel in handling intricate, multidimensional data, offering objective insights, and pinpointing distinctive group-specific disparities (Horst et al., 2019; Dindorf et al., 2021a). Notably, these methods often outperform traditional statistical analysis methods in related databases (Bzdok et al., 2018; Halilaj et al., 2018; Phinyomark et al., 2018). However, their potential is frequently constrained by persistent challenges such as data scarcity.

Data scarcity refers to a situation in which the available data for analysis or decision-making are limited in quantity, quality, or relevance, often presenting challenges in drawing meaningful insights or conclusions (Alzubaidi et al., 2023). Unlike certain fields, such as image classification, which benefit from vast databases containing millions of images (Deng et al., 2009), biomechanical data frequently encounter limitations, typically comprising only hundreds or a few thousand data points (Horst et al., 2021). These limitations stem from various challenges, including difficulties in participant recruitment, resource constraints, ethical considerations, specialized expertise requirements, and the often expensive and intricate nature of the measurements. Consequently, the development and effectiveness of ML algorithms tailored to biomechanical tasks are impeded by the lack of comprehensive datasets.

Data augmentation is a widely used technique in ML and data science, aimed at artificially expanding the size of a dataset by applying various transformations or modifications to existing data (Bicer et al., 2022). The primary objective of data augmentation is to diversify the training dataset, making it more robust, and reducing overfitting (Lashgari et al., 2020). By introducing variations in the data, the model becomes better at generalizing to unseen examples, consequently enhancing its performance on real-world data. The utilization of data augmentation in ML improves a model’s capacity for generalization, which is particularly pronounced in deep learning scenarios (Bicer et al., 2022). For example, in computer vision tasks, data augmentation may encompass randomly rotating or flipping images, changing their color balance, or cropping them differently (Jiang et al., 2020). Similarly, natural language processing techniques can involve paraphrasing sentences, adding synonyms, or introducing typographical errors into the text data (Kang et al., 2021; Bayer et al., 2023).

However, in biomechanics, kinematic data are often presented as tabular or time-series data for dynamic measurements (Horst et al., 2021). In the domain of clinical gait analysis, certain techniques such as magnitude perturbation, temporal perturbation, random rotation, and noise injection have been employed (Kiprijanovska et al., 2020; Tunca et al., 2020; Paragliola and Coronato, 2021). Alternatively, data augmentation for tabular data may involve generating additional samples by interpolating between existing data points or by applying sampling techniques primarily used for imbalanced datasets (for example, the synthetic minority oversampling technique: SMOTE) (Dindorf et al., 2021a; Iglesias et al., 2023).

Furthermore, there exists considerable promise in leveraging generative models for data generation purposes. Generative models such as Variational Autoencoders (VAEs), Generative Adversarial Networks (GANs), and autoregressive models like transformer-based models represent powerful ML models capable of creating new data samples that closely resemble the training data to which they were exposed (Bicer et al., 2022). These models learn the underlying data distributions and generate data points with similar characteristics. This makes them valuable not only for data augmentation but also for content generation (Hussain et al., 2020) and anomaly detection (Yang et al., 2022). Regarding data augmentation, the synthetic data generated by these models can be combined with the original data, resulting in a larger and diversified dataset for training ML models.

Several studies have explored the application of generative models in analyzing human movement data, highlighting the potential of generative models in the biomechanical domain. Researchers have developed (Takeishi and Kalousis, 2021) a generative model for the human gait that ensures physically realistic outputs by integrating a VAE with a differentiable physics engine, demonstrating its efficacy in gait style transfer. Similarly, Liu et al. (2020) employed a conditional GAN to replicate the kinematic attributes of individuals with lateral collateral ligament injuries in their feet and ankles. Additionally, Luo and Tjahjadi, (2020) utilized conditional GANs to create a parametric three-dimensional (3D) model of the human body, including an underlying skeleton, enabling the synthesis of asymmetrical gait samples. Furthermore, Song et al. (2020) harnessed a Deep Convolutional GAN to create binary images that captured three distinct abnormal gait patterns, encompassing falls, reels, and drags.

Although several studies have emphasized the utility of generative AI in the domain of gait data, only one has addressed posture analysis using 3D spinal computed tomography scans of the lumbar spine (Huang and Zhang, 2023). In response to this pressing issue, we explored whether generative AI can bridge the gap in data scarcity by creating synthetic yet realistic stereographic 3D spinal posture data. By leveraging the capabilities of the VAE, we embarked on the task of generating synthetic posture data. The goal is not only to evaluate whether it is possible to train a VAE on posture data and generate synthetic data, but also to scrutinize whether these synthesized postures can be discerned from genuine data by means of Statistical Parametric Mapping (SPM) and a classification task challenging both domain experts and ML classifiers. Furthermore, this study extends beyond data generation. We explored the practical implications of incorporating synthetic data into the learning process. A critical aspect of this inquiry is the use of an autoencoder (AE) for feature learning based on posture data.

AEs are widely used for denoising tasks in clinical biomechanical data. Previous studies (Mohammadian Rad et al., 2018; Elkholy et al., 2019) have demonstrated their effectiveness in improving the discriminative capabilities of models. In various domains, it has been observed that feeding features reconstructed by AEs to a discriminative model as input often yields superior accuracy compared with using the original data (Marchi et al., 2015; Zhao T. et al., 2017; Tu et al., 2020). The latent space of the AE proves to is a valuable resource for automatic feature extraction, a technique that has shown significant utility in other studies (Nguyen et al., 2018; Zaroug et al., 2020; Yang and Yin, 2021). For example, by utilizing latent space in conjunction with other ML models, enhanced performance in various tasks has been demonstrated (Hernandez et al., 2020).

Given the pivotal role of AEs in biomechanical data analysis, enhancing their reconstruction accuracy holds immense value. Consequently, we sought to elucidate whether augmenting the training dataset with generated synthetic postures can lead to reduced reconstruction errors and a more compact feature representation of an AE without sacrificing reconstruction accuracy.

## 2 Materials and methods

**FIGURE 1:**
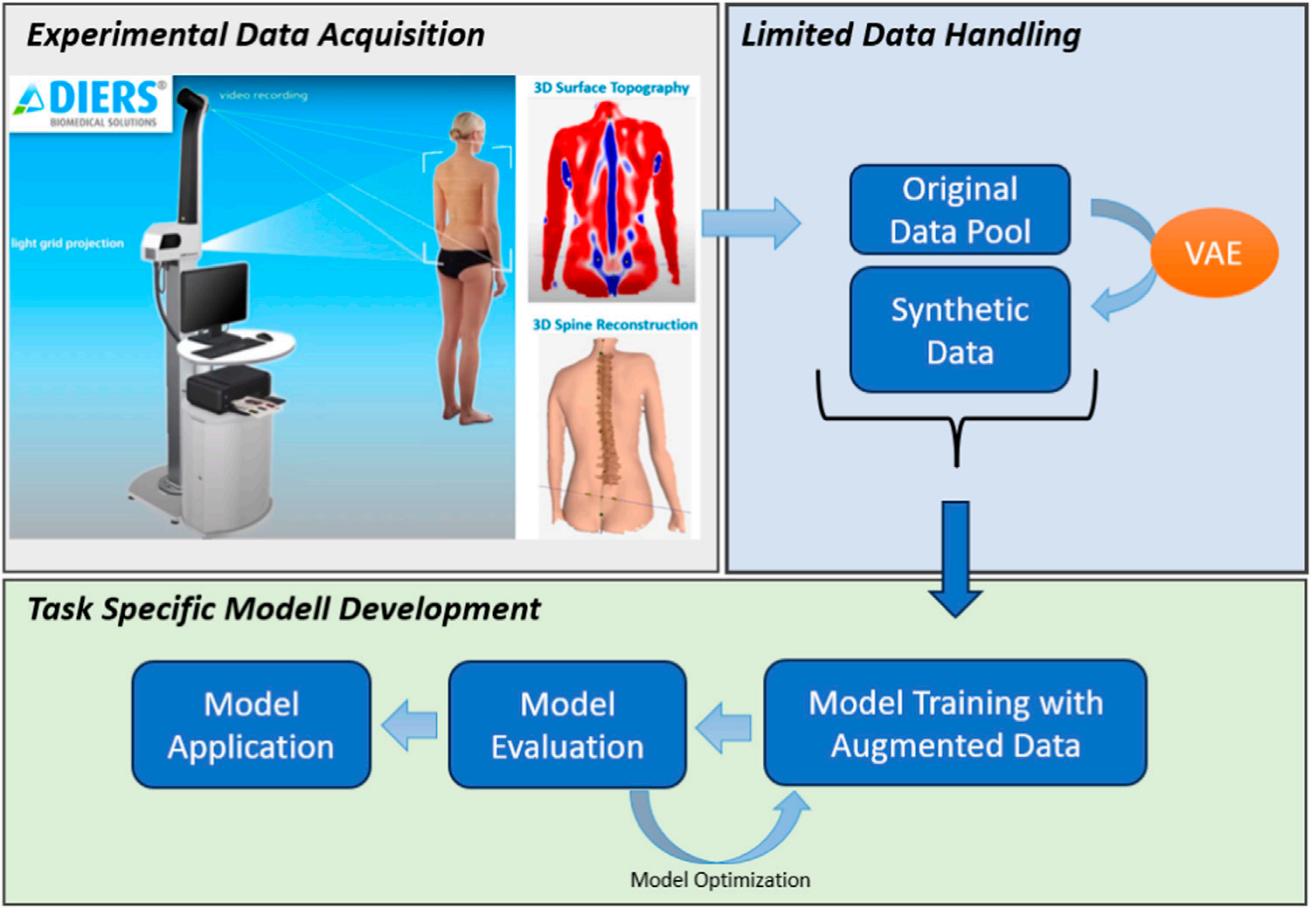
Overall workflow of the study. The top left image illustrates the DIERS formetric III 4D™ system’s (DIERS International GmbH, Schlangenbad, Germany) measurement procedure (originally from (Dindorf et al., 2022b), courtesy of DIERS International GmbH). The original data pool is expanded using a Variational Autoencoder (VAE) (upper right) to address sample size limitations for diverse Machine Learning tasks. This is followed by task-specific model development, exemplified here by a deep Autoencoder, utilizing the augmented data (bottom).

The comprehensive workflow is outlined in Figure 1 for a concise overview. Subsequent sections will furnish detailed insights into each step delineated in the figure.

### 2.1 Subjects and data acquisition

In four separate studies, data were collected from 353 participants. Depending on the study design, as outlined in Table 1, each subject underwent postural data collection for the spine on one or three distinct days. During each session, an average of 12–14 individual images was captured for each subject. This data collection encompassed both healthy individuals and those with various pathologies, such as back pain, spinal fusion, and osteoarthritis. The DIERS formetric III 4D™ system, specifically DICAM v3.7 analyzing software (DIERS International GmbH, Schlangenbad, Germany), was employed as a non-invasive means of rasterstereography, also known as surface topography (ST). Detailed information regarding the participants’ characteristics is presented in Table 1. This method enables comprehensive spinal measurements across all body planes without requiring invasive radiation-based techniques or extensive preparation.

**TABLE 1:** Subject characteristics and related trials.

	Subjects (*n*)	Male (*n*); Female (*n*)	Age (years)	Hight (cm)	BMI (kg/m^2^)	Further information
Healthy ^a^ (asymptomatic)	201	69; 132	41.28 (13.42)	172.51 (8.19)	23.49 (3.21)	18–70 years; free of pain; no history of surgery or fracture between C7 and pelvis; no medical or therapeutic treatment (C7- pelvis) last 12 months; no medical or therapeutic treatment due to musculoskeletal problems (musculoskeletal system except C7-pelvis) last 6 months; BMI ≤30.0; gait stability; an age- and sex-accorded walking speed and spinal function as well as an appropriate joint mobility to theoretically be able to perform a physiological gait pattern; WHO register (INT: DRKS00010834)
Healthy ^b^ (asymptomatic)	25	12; 13	34.68 (12.07)	176.28 (8.83)	24.01 (3.45)	Repeated measurements at three points in time; walking without walking aids and pain; no acute or chronic diseases; no pregnancy; BMI <30; WHO register (INT: DRKS00014325)
Back pain	32	14; 18	44.53 (14.84)	174.00 (11.00)	26.01 (4.79)	Area of pain: 6% thoracic spine (TS), 72% lumbar spine (LS), and 22% TS + LS; no acute fractures, walking restraints, or acute/chronic illnesses that prevent safe walking; WHO register (INT: DRKS00013145)
Spinal fusion	34	20; 14	56.26 (15.40)	171.00 (11.00)	26.95 (4.43)	Spinal fusion somewhere between C7 and L5; no acute fractures, walking restraints, or acute/chronic illnesses that prevent safe walking; WHO register (INT: DRKS00013145)
Osteoarthritis	60	29; 31	64.00 (11.27)	171.00 (9.15)	25.68 (2.35)	30 knee osteoarthritis and 30 hip osteoarthritis; walking without walking aids; no walking impairments that prevent safe walking; no acute or chronic diseases; no pelvic or spinal surgery; no pregnancy; BMI <30; WHO register (INT: DRKS00017240)

^a^ The dataset is part of the dissertation project of Janine Huthwelker. For more details see (Huthwelker et al., 2023).

^b^ The dataset is part of the dissertation project of Friederike Werthmann.

Abbreviations: BMI: body mass index, SD: standard deviations, WHO: world health organization, TS: thoracic spine, LS: lumbar spine, C: cervical, L: lumbal.

We utilized fifty-four static parameters from the system, including measurements such as pelvic obliquity (°), pelvic inclination (dimples) (°), pelvic rotation (°), as well as the orientation of VP, T1–12, and L1–L4 in all planes (°), as part of our modeling process. Supplementary Table S1 provides a comprehensive description of these parameters.

Subsequently, for each participant, we randomly selected three samples without replacement for further calculations. We employed the isolation forest technique (500 trees) to effectively identify and address multivariate outliers. This approach has been demonstrated to be effective in various studies involving kinematic data (Dindorf et al., 2021b; Yee et al., 2021). Consequently, from our initial dataset of 1059 samples, we removed 66 outliers using this method, resulting in a final total of 993 samples, derived from 338 subjects for further analysis.

Although multiple classes of healthy subjects and pathologies were present (Table 1), a single VAE was trained using all the available data. This decision was based on several key considerations.

Insufficient sample sizes were available for each individual class, making it impractical to effectively train separate VAEs for each class.

Previous studies have highlighted the difficulty of discriminating between respective classes, such as distinguishing healthy postures from pathological ones, using ML classifiers (Dindorf et al., 2021b). This suggests that there is limited class-specific information that can be exploited.

Opting for a single VAE offers the advantage of capturing shared patterns and common features that potentially exist across various classes. This approach aims to uncover the underlying similarities that might be overlooked by class-specific models.

By employing a single VAE, the model was designed to learn a universal latent space that remained independent of class labels. This allowed the model to focus on extracting general representations that were common to all classes without being biased by class-specific distinctions.

### 2.2 General workflow and evaluation procedure

**FIGURE 2:**
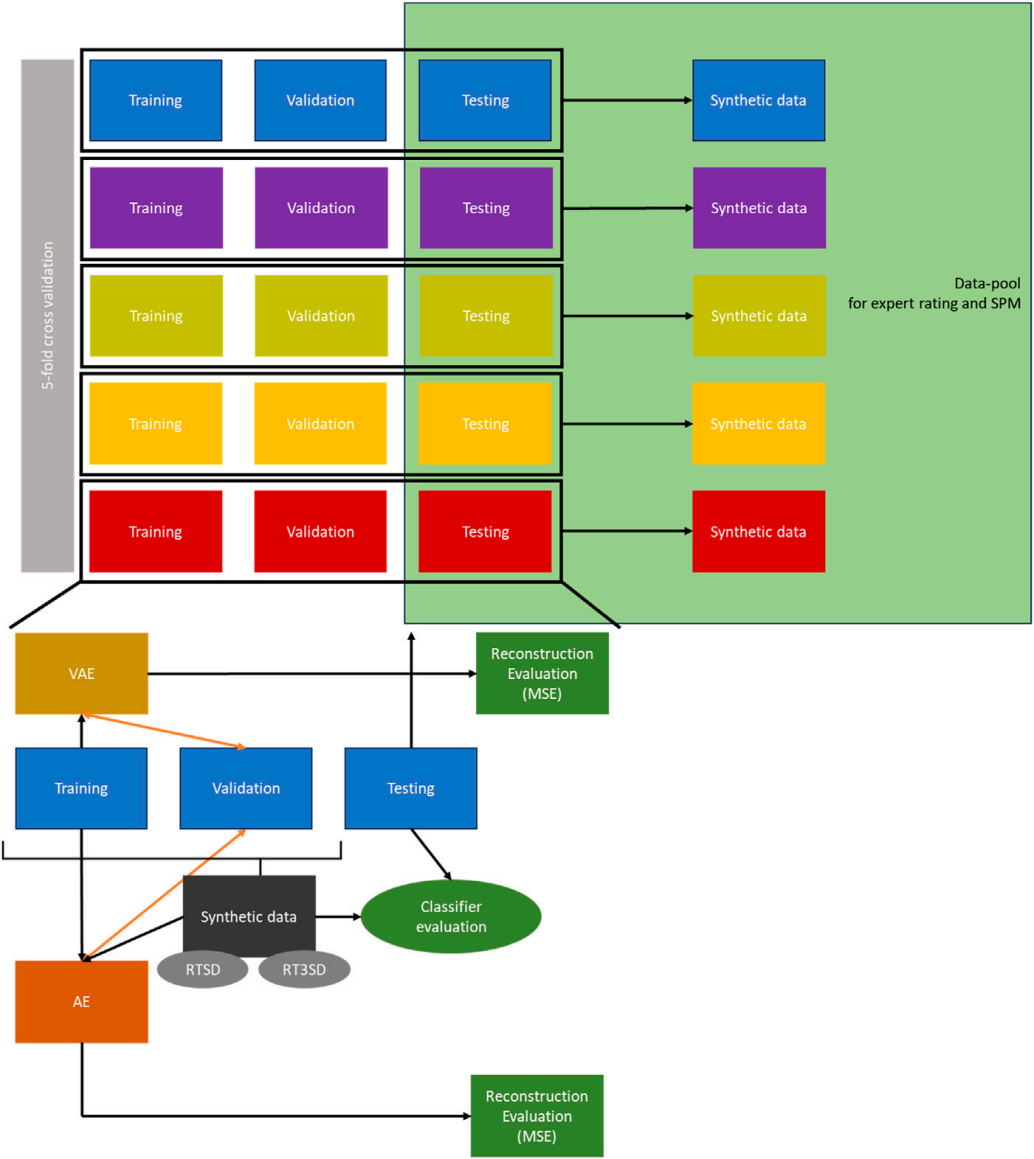
Workflow of the generation, testing and evaluation of the synthetic data. RTSD = dataset with 50% real, 50% synthetic data; RT3SD = dataset with 25% real, 75% synthetic data; MSE = Mean Squared Error; SPM = Statistical Parametric Mapping; AE = Autoencoder; VAE = Variational Autoencoder.

Model development, training, and evaluation of the VAE and AE were integrated into a grouped k-fold cross-validation process (k = 5). In each cross-validation fold, the data underwent random partitioning, with approximately 70% assigned to training, 10% to validation, and 20% to testing (the proportion of test data for each fold is given by k = 5). It was ensured that subject-specific data, considering multiple measurements per subject, remained separate across the sets. The corresponding specific workflow is illustrated in Figure 2. The utilization of grouped k-fold splitting, a method that prevents subject-specific data from being concurrently included in the training, validation, and test sets, offers several advantages. This approach facilitates improved hyperparameter tuning and early detection of overfitting. Furthermore, this method enhances the robustness of the model evaluation by considering the variability across different training instances. Additionally, by ensuring that subject-specific data are not mixed across the training and evaluation sets, it becomes possible to assess how well the models can be generalized to new, previously unseen subjects or data points, thereby providing a more comprehensive evaluation of the model’s performance. The steps pertaining to this workflow are described in detail in the following sections.

### 2.3 VAE implementation

For data generation in our study, we opted for a VAE over a GAN for several compelling reasons. GANs typically require a more extensive and diverse dataset to perform effectively. They thrive when presented with substantial amounts of data that capture intricate patterns and nuances. GANs are sensitive to hyperparameter choices and can suffer from issues such as mode collapse (Saxena and Cao, 2022). In this case, the posture data were not sufficiently extensive to fully harness the potential of the GAN. Our preliminary study, which involved exploratory work with the available posture data, confirmed that VAE outperformed GANs when considering our dataset in terms of both the data quality and stability observed during the training process.

A VAE is an artificial neural network employed for generative tasks. It functions by encoding the input data into a lower-dimensional latent space and then decoding it back into the original data space. The key innovation of a VAE is its ability to model probability distributions in a latent space, allowing it to generate new similar data samples by sampling from these distributions. This makes VAEs particularly useful for tasks, such as data generation, denoising, and representation learning. In short the general information flow in a VAE can described the following (please refer to (Zhao S. et al., 2017) for a detailed description):

The encoder takes input data *x* and produces parameters for a probability distribution over the latent space. Let *z* be the latent variable, *q* (*z*∣*x*) is the approximate posterior distribution, *p*(*z*) is the prior distribution (usually a standard Gaussian), and *μ*(*x*) and *σ*(*x*) are the mean and standard deviation predicted by the encoder. The latent variable z is sampled from the distribution:

$$Z∼N\left(\mu \left(x\right),\sigma {\left(x\right)}^{2}\right)$$

The decoder takes the sampled latent variable *z* and reconstructs the input data *x*. The conditional distribution of the data given the latent variable is modeled as *p* (*x*∣*z*). The reconstructed data *x̂* is sampled from this distribution.

The training objective for a VAE is based on the Evidence Lower Bound (ELBO), which is defined as follows:

$$\text{ELBO}=E_{\left(q\left(z|x\right)\right)}\left[\log p\left(x|z\right)\right]-\text{ KL}\left[q\left(z|x\right)∥p\left(z\right)\right]$$

The first term is the reconstruction term, encouraging the model to generate data similar to the input. The second term is the regularization term, penalizing the divergence between the learned latent distribution *q* (*z*∣*x*) and the prior distribution *p*(*z*).

The information flows from the input data through the encoder to the latent space, and then from the latent space through the decoder to reconstruct the data. The objective during training is to maximize the ELBO, thereby encouraging the model to learn a useful latent representation of the input data.

It aims at a smaller latent dimension than the original number of features to capture the most important features and reduce the complexity of data representation by learning a more compact representation of the data. Furthermore, this proved useful because the smaller latent dimensions acted as a form of regularization, preventing the VAE from overfitting the training data (Mahmud et al., 2020). In addition, it has been suggested that when the latent dimension is smaller, the decoder must generate data with fewer degrees of freedom, which can lead to more coherent and structured generated samples (Zhao et al., 2019).

**FIGURE 3:**
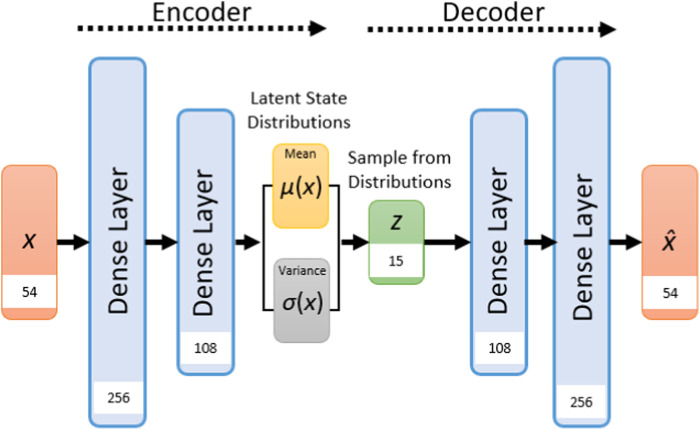
Visualization of the VAE architecture in the current study. The values in the white boxed represent the layer sizes.

To determine the model architecture, a grid hyperparameter search was performed based on the accuracy of the combined losses (reconstruction loss and KL divergence loss) in the validation set. We varied the latent vector length (5, 10, 15, and 20), two hidden layer sizes for the encoder and decoder (54, 108, 256, and 500), batch size (32, 64, and 128), learning rate (0.01, 0.001, and 0.0001), and number of epochs (200, 400, 600, and 1,000). The VAE model employs an Adam optimizer to minimize the combined loss function. Based on each training set, scaling was applied using StandardScaler from Scikit-learn (Pedregosa et al., 2011). The final model has the following configuration:

The encoder network operated on the input posture data (shape: 54) through two dense layers with specific sizes of 256 and 108, utilizing both Rectified Linear Unit (ReLU) activation functions. These layers reduced the input data to a latent space of 15 dimensions. This is followed by a symmetric decoder section comprising two corresponding dense layers, both employing ReLU activation, and an additional final layer employing linear activation. The epochs were set to 400 with a learning rate of 0.001, and a batch size of 128. For a visual representation of the architecture of the VAE please refer to Figure 3.

Although the intermediate losses employed during VAE training are pivotal for the training process, they may not be as informative or comparable. Instead, we report the Mean Squared Error (MSE) to evaluate the reconstruction errors and conduct model comparisons.

### 2.4 Evaluation synthetic data

To evaluate the distinguishability of synthetic data from real data, we adopted three distinct approaches: (a) judgment by domain experts, (b) implementation of an ML classifier, and (c) statistical evaluation using SPM.

**FIGURE 4:**
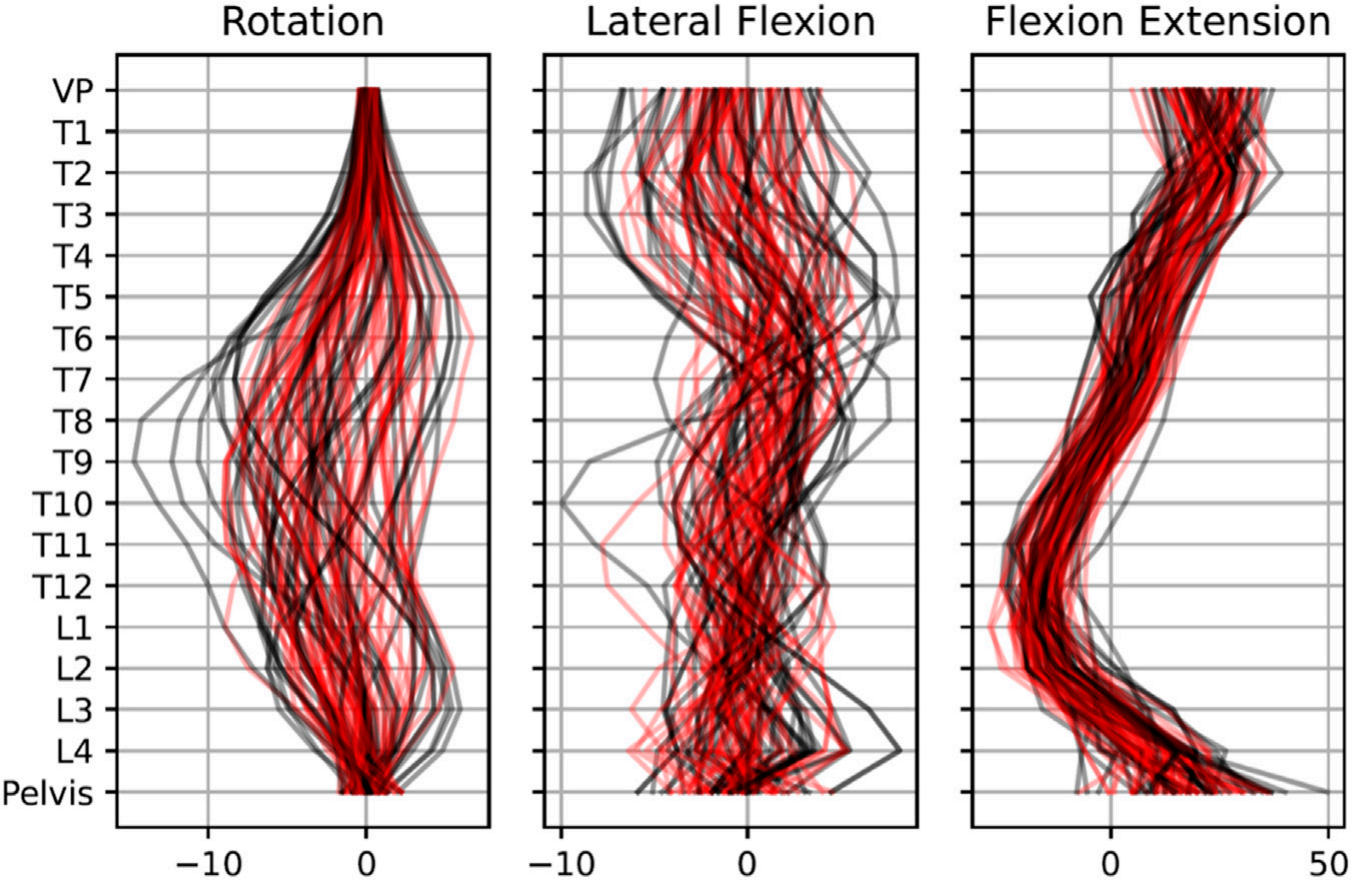
Visual comparison of 50 exemplary real (black line) and 50 exemplary synthetic (red line) data samples. Data are rescaled to original feature space.

First, we generated synthetic data for each VAE model during cross-validation of the required size (see below). Therefore, random sampling from a standard Gaussian distribution was performed to generate latent vectors. These latent vectors are then passed through the decoder component of the trained VAE model. Subsequently, we combined the original data from the test set with synthetic data, enabling us to perform the identification tasks denoted as (a), (b), and (c). For expert-based evaluation (a) and SPM analysis (c), we rescaled the feature values to match the scale and distribution of the original data. This was done to ensure that the experts could assess the data in an accustomed manner while preserving the fidelity of their evaluation process:

a) In the expert-based evaluation, we opted for a random subset of 100 real and 100 synthetic data samples because a comprehensive assessment was economically infeasible due to constraints on the experts. Therefore, for each fold, we randomly selected and combined ten real samples from the test set with ten synthetic samples generated by the respective VAE model. Each sample underwent an independent evaluation by three experts, and the final expert-based classification was determined via a majority vote. These experts possessed extensive experience working with spinal data and were familiar with the dataset. During the evaluation, the data were presented visually, similar to the illustration in Figure 4. The expert ratings were organized, and the accuracy for each rater and across all ratings was calculated using MATLAB (MathWorks, Natick, Massachusetts, United States). The loose majority voting was calculated based on (Ballabio et al., 2019). Fleiss’ kappa was calculated using the SPSS software (IBM, Armonk, New York, United States).

b) We conducted a supervised classification task to discriminate between real (all test set samples) and synthetic samples equal in size to the test set. To achieve this, we employed a k-nearest neighbour classifier with k = 10. The other parameters were set to the default scikit-learn parameters (Pedregosa et al., 2011). To gauge the effectiveness of the classifier in distinguishing between the two data types, we leveraged the cross-validation accuracy score derived from a 5-fold cross-validation procedure.

c) For further evaluation of the synthetic data based on (Bicer et al., 2022) the statistical difference between the synthetic and real data for each vertebrae in the anatomical plane were compared employing a non-parametric 1D two-tailed unpaired *t*-test (*α* = 0.05) using the spm1d package (Pataky et al., 2013) in MATLAB. Hence, in the actual dataset, a single sample was randomly chosen for each subject. A synthetic dataset of equal size (n = 338) was created by randomly selecting synthetic samples generated during the cross-validation folds.

### 2.5 Use case evaluation AE

For present studies that use case evaluation, the primary emphasis should be dimensionality reduction. We do not focus on the generative capabilities or probabilistic modeling offered by VAEs. Our objective is to establish a deterministic mapping from the input data to a latent representation, ensuring that similar input data points are consistently mapped to similar points in the latent space without introducing any randomness. To satisfy these criteria, we chose to utilize an AE because it does not introduce a probabilistic element that could result in variations within the latent-space representations. Second, AEs are simpler to implement and incur less computational overhead. Unlike VAEs, AEs do not require complex probabilistic modeling or variational inference techniques.

We evaluated the potential usefulness of artificially created posture data using the VAE training of the AE in three different scenarios:

• Utilizing only the unaugmented data as training data, referred to as *RTD* (100% real training data).
• Employing the real data combined with synthetic data in equal proportions in the training dataset, denoted as *RTSD* (50% real, 50% synthetic).
• Expanding the real data with synthetic data three times its size, labelled as *RT3SD* (25% real, 75% synthetic).

For augmented data generation, we randomly selected one trained VAE model that resulted from the cross-validation process and created synthetic data of the respective sizes, as described in the previous section.

The AE was trained during grouped k-fold cross-validation, similar to the training of the VAE (k = 5), to assess how well the AE could generalize its learned representations to new, previously unseen subjects. Scaling was applied based on each training set (without synthetic data) using StandardScaler from Scikit-learn.

Similar to VAE, a grid hyperparameter search guided by the validation set accuracy using unaugmented data was performed. The latent dimension was set to be equal to that of the VAE, and the number of hidden layers was set to three. The hidden layer sizes (25, 50, 100, 250, and 500) of the encoder and decoder, batch sizes (32, 64, and 128), and learning rates (0.01, 0.001, and 0.0001) were varied. Early stopping was integrated into the training procedure, which involved monitoring the validation loss and restoring the best weights when necessary, with a patience setting of 10 epochs and a maximum of 1,000 epochs. This approach led to the final deep AE configuration as follows:

The model was structured with an encoder section featuring three dense layers (500, 250, and 50 units in the first, second, and third layers, respectively), which collectively reduced the input data into a 15-dimensional latent space. This was followed by a symmetric decoder section consisting of three corresponding dense layers. All of these layers utilize ReLU activation functions, except for the final layer of the encoder and decoder, which employs a linear activation function. To train the AE, we employed the MSE loss function in combination with the Adam optimizer (learning rate = 0.001) and a batch size of 64.

Finally, we explored the potential for reducing the latent dimension while maintaining the same reconstruction accuracy as in the unaugmented data by augmenting the training data while preserving other hyperparameters. This exploration was guided by a manual search procedure that considered the accuracy of the validation set.

### 2.6 Statistics and further calculations

Modeling was implemented using the TensorFlow (Abadi et al., 2016) and Keras (Chollet, 2015) frameworks. Visualization was performed employing matplotlib (Hunter, 2007). Visual exploration of the latent space was performed with Uniform Manifold Approximation and Projection for Dimension Reduction (UMAP) (McInnes et al., 2018).

## 3 Results

### 3.1 VAE and synthetic data evaluation

**FIGURE 5:**
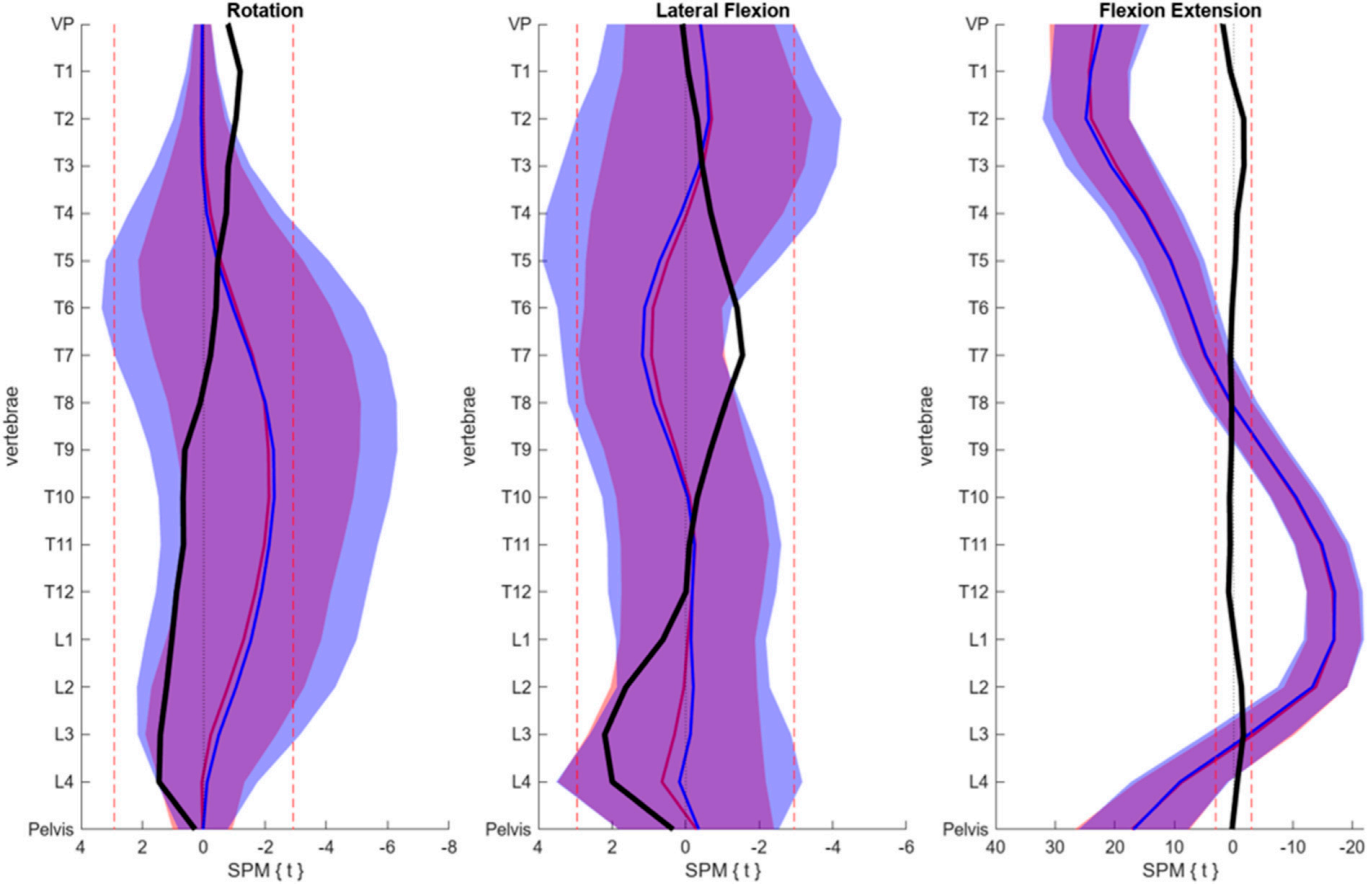
SPM results displayed for each anatomical plane. The blue line and area each represent the mean and SD of the real data, while the red line corresponds to synthetic data. The red dotted lines indicate the critical t-values, signifying the absence of significant differences in-between. Additionally, the black line depicts the t-values observed for each vertebra.

The reconstruction errors of the trained VAE are listed in Table 2. Subsequently, the trained VAE was employed to generate synthetic data. Both generated synthetic data samples as well as real posture data samples are visually presented and compared alongside each other in Figure 4. Notably, there were no discernible systematic differences between the real and synthetic data when viewed visually. This was also statistically confirmed by the SPM, which showed that for no vertebrae, the difference between the real and synthetic data was significant (Figure 5).

**TABLE 2:** MSE results for the VAE and AE for each cross-validation fold, as well as mean and SD (bold values) over all folds. Data: RTD = 100% real training data; RTSD = 50% real, 50% synthetic; RT3SD = 25% real, 75% synthetic.

Fold	VAE			AE (latent dim. 15)						AE (latent dim. 7)					
				RTD		RTSD		RT3SD		RTD		RTSD		RT3SD	
	Train MSE	Val. MSE	Test MSE	Train MSE	Test MSE	Train MSE	Test MSE	Train MSE	Test MSE	Train MSE	Test MSE	Train MSE	Test MSE	Train MSE	Test MSE
1	0.01	0.14	0.15	0.02	0.17	0.01	0.01	0.01	0.01	0.17	0.34	0.10	0.07	0.05	0.06
2	0.01	0.13	0.13	0.07	0.19	0.01	0.02	0.01	0.02	0.18	0.33	0.07	0.08	0.11	0.12
3	0.01	0.11	0.13	0.04	0.18	0.01	0.02	0.01	0.01	0.18	0.30	0.08	0.08	0.10	0.09
4	0.01	0.10	0.12	0.01	0.15	0.01	0.01	0.01	0.01	0.14	0.35	0.09	0.08	0.06	0.06
5	0.01	0.18	0.11	0.01	0.14	0.01	0.09	0.01	0.09	0.17	0.29	0.03	0.39	0.04	0.37
**Mean**	**0.01**	**0.13**	**0.13**	**0.03**	**0.17**	**0.01**	**0.03**	**0.01**	**0.03**	**0.17**	**0.32**	**0.07**	**0.14**	**0.08**	**0.14**
**SD**	**0.00**	**0.03**	**0.01**	**0.03**	**0.02**	**0.00**	**0.03**	**0.00**	**0.04**	**0.02**	**0.02**	**0.03**	**0.14**	**0.03**	**0.13**

The results of the ML and expert-based evaluations assessing the separability of real and synthetically generated posture data using the VAE are presented in Table 3. Both the ML classifier and human experts struggled to accurately distinguish between synthetic and real data, with experts exhibiting a notably poorer performance than the ML classifier.

**TABLE 3:** Separability as classification results or real and synthetic posture data comparing experts and human performance.

Accuracy		ML evaluation		Human experts’ evaluation	
		66.53% ± 2.72%		52.17% (*κ* = .073)	
		Actual real	Actual synthetic	Actual real	Actual synthetic
Confusion Matrix	Predicted real	562	234	160	147
	Predicted synthetic	431	759	140	153

The first rater’s accuracy was 52.00%, the second one achieved 51.00%, and the third rater rated 53.50% of all cases correctly. The interrater reliability is calculated at κ = .073 indicating that only slight agreement between the raters (Landis and Koch, 1977). Loose majority vote (50%) shows data was more often rated as real (real = 307, synthetic = 293).

### 3.2 Use case evaluation AE

The reconstruction errors for the AE and the real and augmented datasets are listed in Table 2. An evident enhancement of more than five times in the accuracy of the test set reconstruction becomes strikingly apparent when the training data are expanded with synthetic data using the VAE. This improvement was particularly prominent when using synthetic data of equal proportions in training (RTSD). Extending the original data to three times its size (RT3SD) only slightly reduced the test-set reconstruction error.

**FIGURE 6:**
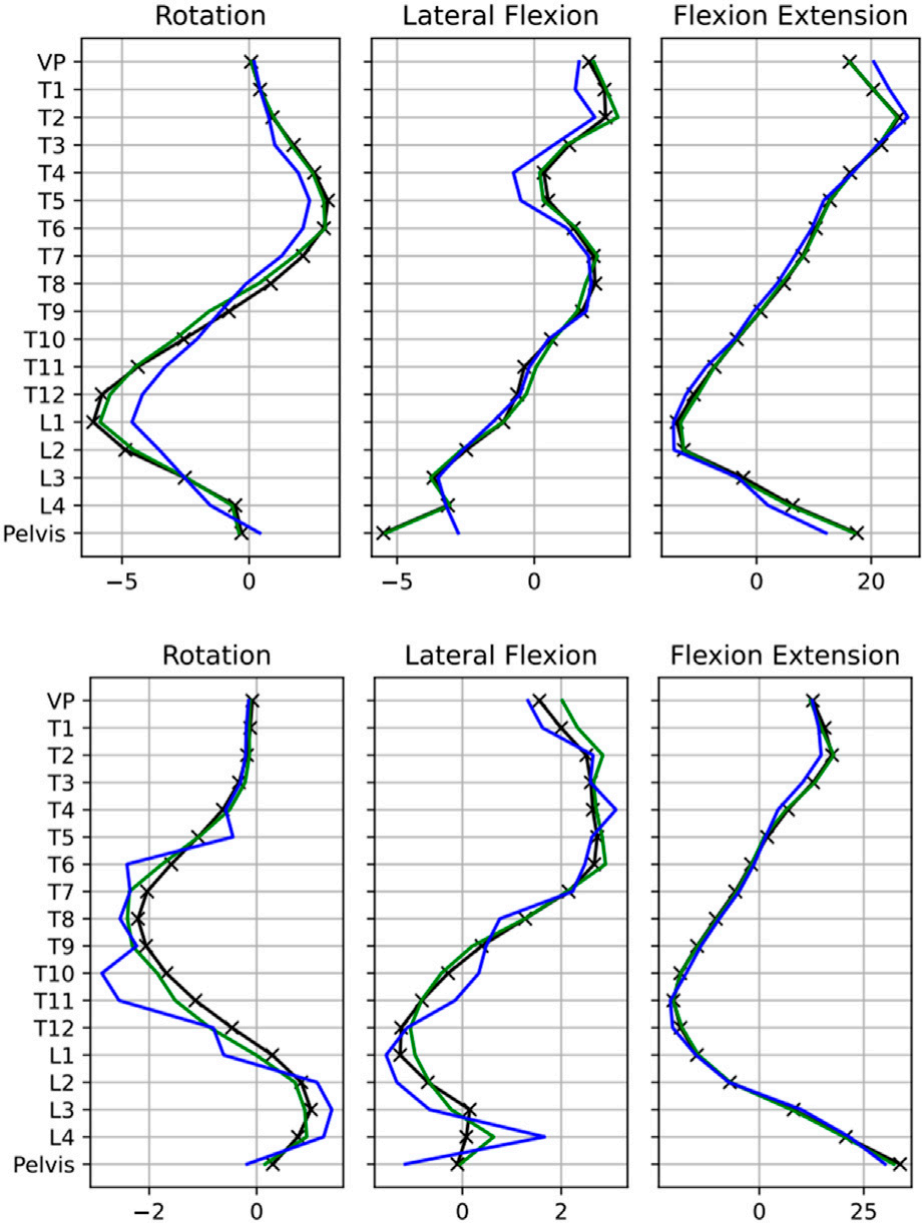
Comparison between actual (represented by black lines with markers) and reconstructed data. The blue lines show reconstructions using solely the original training data, while the orange lines denote reconstructions based on training data augmented by synthetic data (RTSD) generated through the VAE for exemplary four subjects Data is rescaled to original feature space.

The impact of this augmentation on the reconstruction quality becomes apparent when visually comparing the performance of the AE with and without the inclusion of synthetic data. This comparison demonstrates the superior reconstruction with the augmented dataset (see Figure 6).

Explorative reduction of the latent dimension from 15 to seven while keeping the other hyperparameters leads to a slightly better reconstruction performance of the AE while training with the augmented data compared to training only on the unaugmented data with a latent space of 15. In contrast, when using only the unaugmented data RTD with three latent dimensions, the performance deteriorated significantly.

**FIGURE 7:**
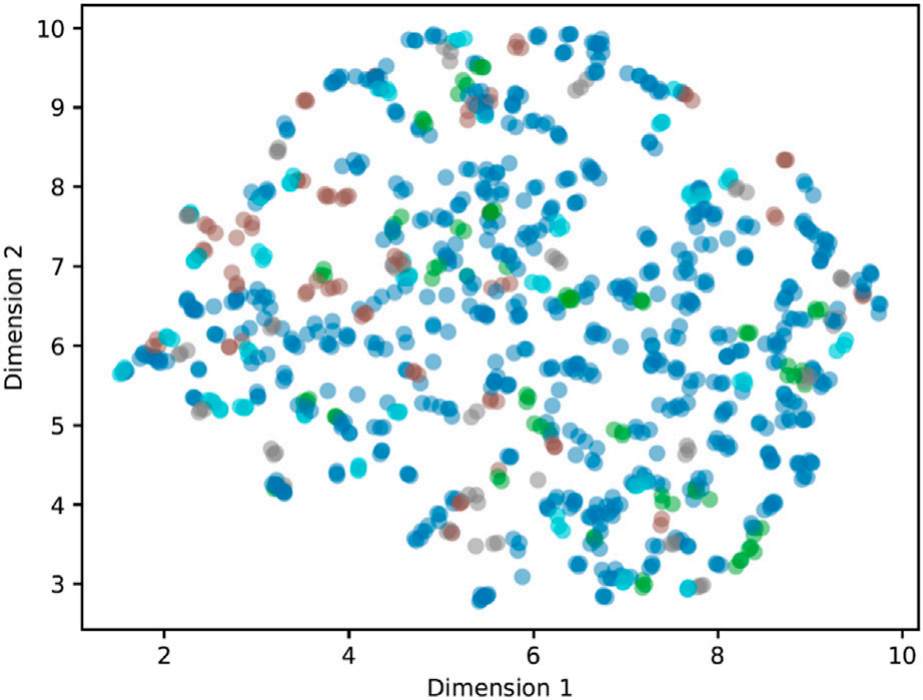
Latent space visualization using UMAP (McInnes et al., 2018) for a latent dimension of 15 and training using the augmented data RT3SD. The color code represents the class membership according to the datasets used (see Table 1).

Visualization exploration of the latent space using UMAP (McInnes et al., 2018) (Figure 7) shows no clearly visible clusters and no clear grouping of the datasets used for the study (healthy, back pain, spinal fusion, osteoarthritis).

## 4 Discussion

This study addresses a critical issue in the field of biomechanics: scarcity of data for the development of ML models. Our exploration of the use of generative AI to generate synthetic posture data offers promising insights into how limited data challenges can be mitigated and how biomechanical ML can be enhanced.

The promising results regarding loss reduction, as well as the low MSE values for data reconstruction, indicate the VAE’s ability to capture the underlying features of the data distribution and show that it is generally possible to develop a VAE model on posture data. Our results align with those of recent biomechanical studies that have successfully applied Variational VAEs to capture essential data distribution features (Huang and Zhang, 2023; Kneifl et al., 2023).

Addressing the quality of synthetic data is of pivotal concern when it is applied to ML tasks. The synthetic data closely mirror the characteristics of the real data (Sharifi Renani et al., 2021). However, evaluating the quality of the synthetic data in the absence of a definitive benchmark dataset is challenging. Although various quantitative metrics have been suggested (Zhou et al., 2019), their applicability in the biomechanical context remains limited (Bicer et al., 2022). To overcome this challenge, we adopted a comprehensive evaluation approach for synthetic data, encompassing both objective assessments through ML classification and SPM and subjective evaluations through expert ratings.

Visually, the synthetic data closely resemble the real data. On a statistical basis, employing SPM, no discernible differences were detected between the real and synthetic data. Moreover, when evaluated by both ML classifiers and domain experts, distinguishing between real and synthetically generated posture data proved highly challenging. The experts exhibited minimal-to-negligible consensus, underscoring the inherent challenges of such assessments. This multifaceted evaluation collectively indicates that the synthetic data generated by the VAE exhibit a high level of quality and maintain consistency with the real-world posture data. Consequently, it can be concluded that the proposed VAE is highly effective for generating synthetic posture data that accurately emulate real data.

Incorporating synthetically generated posture data into the ML process, here with the use case example of an AE, yielded notable improvements in training and test set reconstruction accuracy. This is in line with several studies that demonstrated that AE benefits from larger datasets (Zhao et al., 2015). When incorporating synthetic data, a remarkable improvement in the accuracy of the test set reconstruction became evident, with a more than seven-fold reduction in the test set MSE compared with using unaugmented data for training. These results suggest that augmenting the training data for training an AE with synthetic examples by means of a VAE not only enhances the model’s ability to reconstruct the data it was trained on but also improves its generalization to unseen test data, which has also been reported in other works (Wan et al., 2017; Kornish et al., 2018).

It is important to note that alternative approaches to data augmentation have the potential to enhance model performance when dealing with limited data. For instance, transfer learning, an ML technique, allows a model to leverage the knowledge gained from a previous task to enhance its generalizability to a new task. Transfer learning compensates for the scarcity of labeled data by transferring knowledge from other well-labeled data sources. To address the shortage of abnormal gait data, researchers have (Pandit et al., 2019; Martinez and Leon, 2020) employed various neural networks pretrained on extensive datasets. One approach of interest could involve combining transfer learning with subsequent training on augmented data.

Visual exploration of the latent space revealed a notable absence of distinct clusters and clear groupings among the datasets used in this study, encompassing postures categorized as healthy and those associated with back pain, spinal fusion, and osteoarthritis. This finding underlines the challenges in discriminating between healthy and pathological postures, a hurdle that previous research has highlighted when employing ML classifiers without the benefit of feature learning techniques (Dindorf et al., 2021b). Considering these challenges, the findings of this study are comprehensible and contribute to the current state of research by demonstrating that even the application of feature learning through an AE does not yield a discernible enhancement in discriminability.

Notably, our VAE was not trained separately for each class for the aforementioned reasons, which may have resulted in a mixed latent space in which class-specific information was not well separated. Consequently, class-specific discriminative characteristics may not be as pronounced in the synthetic data, potentially impeding the formation of discernible clusters. Future research should consider including dynamic movement data from the spine as a promising direction. The dynamic aspects of posture and movement could potentially offer more distinctive class differences, potentially facilitating the identification of clusters; hence, there is significant inter-subject variability in spine movement, for example, during gait (Prost et al., 2021). In the context of distinguishing between biological sexes, recent findings have indicated a significant improvement in classification accuracy when utilizing dynamic data as opposed to relying solely on static data (Dindorf et al., 2021c). This highlights the potential of using dynamic data to enhance the accuracy of classification models for specific applications.

Although our research has yielded promising insights into the use of generative AI to address data scarcity in biomechanical ML, it is crucial to acknowledge several limitations that should be considered when interpreting the results and planning future studies. Despite the favorable results in distinguishing synthetic data from real data, it is important to mention that there may still be subtle differences between the two. Synthetic data, although visually and quantitatively similar, may not capture all of the intricacies of real-world biomechanical postures, potentially leading to limitations in specific applications where extreme precision is required.

This study primarily relied on a specific dataset obtained from a particular group of subjects via surface topography. The effectiveness of the generative AI approach may vary when applied to different biomechanical datasets or to data collected using diverse measurement techniques. The ability of the model to be generalized to broader and more diverse populations requires further investigation.

Although our results demonstrate the benefits of augmenting the training dataset with synthetic data, the optimal balance between real and synthetic data remains an open question. The study could only show that with the current AE expanding the real data with synthetic data to three times their size (RT3SD) slightly improved the reconstruction performance compared to real data combined with synthetic data of equal proportions (RTSD). Further research is required to explore the potential impacts of varying proportions of synthetic data.

The use of synthetic data in healthcare raises ethical concerns. On one hand, it mitigates privacy risks by minimizing the demand for additional patient data, thereby reducing the risk of data breaches. However, synthetic data may not fully represent the complexities of actual patient data, potentially leading to biased or inaccurate outcomes. The extent to which accountability applies, in this case, must be discussed in a context-specific manner.

Future directions may involve extending the application of generative AI to other biomechanical domains such as dynamic spinal data. An increase in the volume of accessible posture data has the potential to significantly enhance the applicability of GANs. Therefore, a future comparative analysis between GANs and the approach presented in this study, if feasible, is considered important. Additionally, investigating the impact of synthetic data on various ML architectures or distinct tasks, such as regression or classification, is a promising area of research. In the context of gait data, deep generative models combined with differentiable physics engines have been proposed to ensure that the generated data are in line with physical laws (physically informed modeling) (Takeishi and Kalousis, 2021). The adoption of this methodology in the context of posture data could ensure the realism of the generated data and should be evaluated in future studies. Furthermore, an intriguing direction for future research could be the exploration of an extended VAE that conditions data generation or reconstruction on additional information, such as class labels, or other attributes, such as biological sex. This exploration is particularly relevant as existing studies highlight the presence of biological sex differences in spinal data (Yukawa et al., 2018; Mohan and Huynh, 2019; Ludwig et al., 2023). These models, known as Conditional Variational Autoencoders (CVAEs) (Zhao T. et al., 2017), can accentuate the class membership, potentially leading to the generation of more realistic posture data by incorporating additional subject characteristics. To the best of our knowledge, this application has not been explored in the biomechanical domain.

## 5 Conclusion

In summary, our study underscores the potential of generative AI, specifically VAEs, in addressing data-scarcity challenges within the biomechanics field. By generating synthetic posture data that closely mirror real-world observations, our study presents a viable approach path for expanding datasets, strengthening model performance, and advancing biomechanical applications.

## Data Availability

The datasets for this article are not publicly available due to concerns regarding participant/patient anonymity. Requests to access the datasets should be directed to the corresponding author.
