# Supplementary material for: Enhancing biomechanical machine learning with limited data: generating realistic synthetic posture data using generative artificial intelligence
Source: Front Bioeng Biotechnol. 2024 Feb 14;12:1350135. doi: 10.3389/fbioe.2024.1350135 (PMC10899878; doi:10.3389/fbioe.2024.1350135)
Supplement: Supplementary file 1 [file Table1.pdf]

Table Sub1. Variables used for modeling: Variables were measured in sagittal, frontal, and transversal plane. The descriptions were adapted from the DIERS manual.

**Variable**	**Plane**	**Description**
VP/C7 (Vertebra prominens/7. cervical vertebral body)<br><br>T1–T12<br><br>(Thoracic spine)<br><br>L1–L4<br><br>(Lumbar spine)	Sagittal:<br><br>Vertebral sagittal Flexion and Extension (°)	The parameter describes the inclination of the calculated vertebra in space (relative to a plumb/gravity line), as seen from a left view. The angle (°) is calculated from the projection of the vertebra in a sagittal plane (rotation and lateral flexion are ignored). A positive value implied a forward tilt of the vertebra (flexion).
	Frontal:<br><br>Vertebral Lateral Flexion (°)	The parameter describes the lateral inclination of the vertebra in space (relative to a plumb/gravity line), as seen from a posterior–anterior view. The angle (°) is calculated from the projection of the vertebra in the coronal plane (rotation and sagittal extension/flexion are ignored). A positive value indicates a tilt of the vertebra to the left (lateral flexion left).
	Transversal:<br><br>Vertebral Rotation (°)	The vertebral rotation describes the rotation of a vertebra in the transversal plane (relative to the neutral pelvis). A positive value signifies a vertebra is rotated to the left (counterclockwise) when seen from behind.<br><br>The rotation of vertebral bodies happens in situ and, therefore, the rotational direction between the surface and vertebral rotation changes. Hence, a surface rotation to the right, mathematically represented with a +, becomes a vertebral body rotation to the left. This is due to the calculation process in which a vector is used that points from the Processus spinosus towards the middle of the vertebral body, indicating that the surface rotation changes its direction within the vertebral body.
Pelvis	Pelvic Obliquity (°)	A line is drawn from DL to DR (left and right dimple), and is compared to a horizontal line representing the horizon. The angle (°) between them is measured, with a positive value signifying that the right pelvis is elevated.
	Pelvic Inclination (dimples) (°)	The parameter describes the mean vertical torsion of the two surface normals on DL and DR.

	Pelvic Rotation (°)	The pelvic rotation is the rotation in the transversal plane of the right dimple relative to a reference coronal plane (that is defined from the system setup), perpendicular to the camera-projection axis. A positive value implies that the pelvis is rotated to the left when seen from behind (the value is corrected * (−1)).
